# Ophiopogonin D, a Steroidal Glycoside Abrogates STAT3 Signaling Cascade and Exhibits Anti-Cancer Activity by Causing GSH/GSSG Imbalance in Lung Carcinoma

**DOI:** 10.3390/cancers10110427

**Published:** 2018-11-08

**Authors:** Jong Hyun Lee, Chulwon Kim, Seok-Geun Lee, Gautam Sethi, Kwang Seok Ahn

**Affiliations:** 1 Department of Science in Korean Medicine, College of Korean Medicine, Kyung Hee University, 24 Kyungheedae-ro, Dongdaemun-gu, Seoul 02447, Korea; mirue88@nate.com (J.H.L.); sunny10526@nate.com (C.K.); seokgeun@khu.ac.kr (S.-G.L.); 2 Department of Pharmacology, Yong Loo Lin School of Medicine, National University of Singapore, Singapore 117600, Singapore

**Keywords:** ophiopogonin D, STAT3, ROS, GSH, apoptosis, NSCLC

## Abstract

Natural medicinal plants are multi-targeted in nature and their anti-cancer activities are also complex and varied, thus requiring a more systematic analysis of their modes of action. Since the activation of signal transducer and activator of transcription 3 (STAT3) is often deregulated in non-small cell lung carcinoma (NSCLC) cells and tissue specimens, its negative regulation can form the basis for identification of targeted therapy. In this report, we analyzed the possible anti-cancer effects of ophiopogonin D (OP-D) and the underlying mechanisms by which OP-D exerts its actions in NSCLC. OP-D exhibited substantial suppressive activity on STAT3 signaling and this effect was found to be mediated via oxidative stress phenomena caused by disturbance in GSH/GSSG ratio. In addition, OP-D induced apoptosis, activated caspase mediated apoptotic cascade and decreased expression of various oncogenic genes. Consistently, OP-D treatment significantly reduced NSCLC tumor growth in preclinical mouse model with via decreasing levels of p-STAT3. OP-D was also found to attenuate the expression of STAT3-regulated anti-apoptosis, cell cycle regulator, and angiogenesis biomarkers. Our findings suggest that OP-D can induce apoptosis and exert anti-tumor effects by inhibition of STAT3 signaling pathways in NSCLC.

## 1. Introduction

Apoptosis is an important cellular process which is marked by cell shrinkage, chromatin condensation, and the formation of DNA fragmentation [1]. Apoptotic cascade may be mediated via an intrinsic pathway in which the cell can destruct itself because it can sense cellular stress, while in the extrinsic pathway it is killed because of signals perceived from external factors [2]. The two pathways can require the actions of both initiator as well as executioner caspases to implement the apoptotic cell death [3]. The ability to regulate cellular life or death can also have tremendous therapeutic implications.

Signal transducer and activator of transcription 3 (STAT3) is a critical transcription factor [4,5,6] that regulates cellular apoptosis, proliferation, and metastasis by the modulation of varied gene expression [7,8]. It has been found to be persistently activated in diverse malignancies [9,10,11,12,13,14,15,16,17,18] as well can also be induced by a variety of agents that can lead to its dimerization and nuclear translocation [4,19,20,21,22,23]. Specifically, STAT3 is activated after phosphorylation of tyrosine 705 upon interacting with a ligand and can also occur through phosphorylation of serine 727 by mitogen-activated protein kinases (MAPK) and activation induced through JAKs and c-Src tyrosine kinases [24,25,26]. Activation of STAT3 can contribute significantly to increased proliferation, survival, angiogenesis, and metastasis [16,27,28,29]. At present, STAT3 inhibitors including natural compounds, peptides and oligonucleotides have been developed and are being clinically established [4,5,30], but identification of efficacious agents targeting this protein still remains an important challenge.

*Ophiopogon* root extract is extracted from the root tuber of *Ophiopogon japonicus* (Thunb.) Ker-Gawl (family Liliaceae). It encompasses multiple active ingredients saponin such as *ophiopogon japonicus saponin* (ophiopogonin) A, B, B’, C, C’, D, D’, and methylophiopogonanone. It also contains various volatile oils and among these oils, ophiopogonin D (OP-D) has been reported to exhibit significant anti-inflammatory and antioxidant effects [31]. Prior studies have reported that OP-D can protect cardiomyocytes against doxorubicin-induced injury [32] and attenuate doxorubicin-induced autophagy by reducing mitochondrial damage [33]. In addition, OP-D has been reported to exhibit diverse pharmacological actions, including attenuation of venous thrombosis [34], reduction of inflammation [35], as well as antitussive activity.

Our group has previously reported that OP-D could induce apoptosis by blocking NF-B and Akt signaling pathways and could also enhance the apoptosis-inducing effect of chemotherapy [36]. In the present report, we noticed that OP-D can modulate STAT3 activation and abrogate tumor growth in a preclinical non-small cell lung carcinoma (NSCLC) model.

## 2. Results

### 2.1. OP-D Down-Modulates Phospho-STAT3 Expression and DNA Binding Capacity in NSCLC Cells

**Figure 1:**
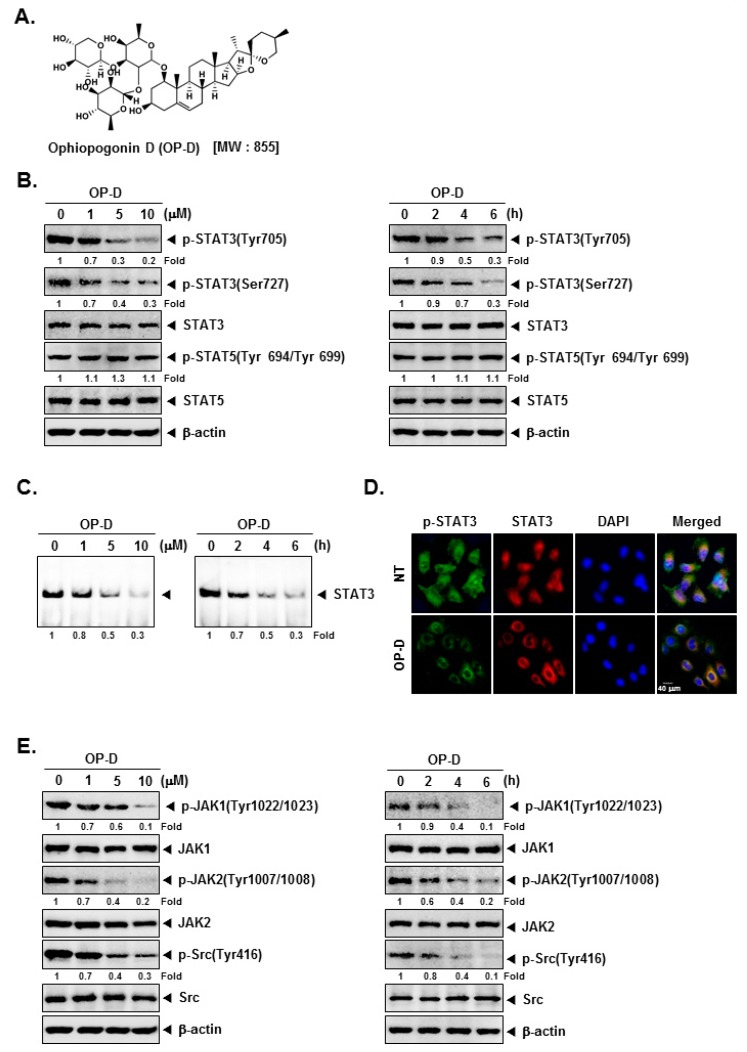
Ophiopogonin D (OP-D) blocks the Stat3/Jak signaling pathway. (**A**) The chemical structure of OP-D. (**B**) A549 cells were treated with the indicated concentrations of OP-D for 6 h (left panel) and treated with 10 µM of OP-D for various time intervals (right panel). Thereafter, equal amounts of lysates were analyzed by Western blot analysis using various antibodies. (**C**) A549 cells were treated as described above and electrophoretic mobility shift assay was done. (**D**) A549 cells were treated with 10 µM of OP-D for 6 h and then analyzed for the intracellular distribution of by immunocytochemistry. (**E**) A549 cells were treated as described above in panel A and Western blot was performed using various antibodies.

We first determined the effect of OP-D on constitutive STAT3 activation in NSCLC cells. We noticed that OP-D substantially reduced constitutive STAT3 activation at both tyrosine 705 and serine 727 residues in a concentration- and time-dependent manner. Interestingly, OP-D had a minimal effect on the expression of STAT5 (Signal transducer and activator of transcription 5) activation (Figure 1B). EMSA (Electrophoretic mobility shift assay) analysis of nuclear extracts showed that OP-D can also substantially suppress STAT3 DNA binding ability (Figure 1C). OP-D also reduced the nuclear levels of p-STAT3 and STAT3 as shown in Figure 1D.

### 2.2. OP-D Modulates the Phosphorylation of Oncogenic Kinases and Inducible STAT3 Activation

**Figure 2:**
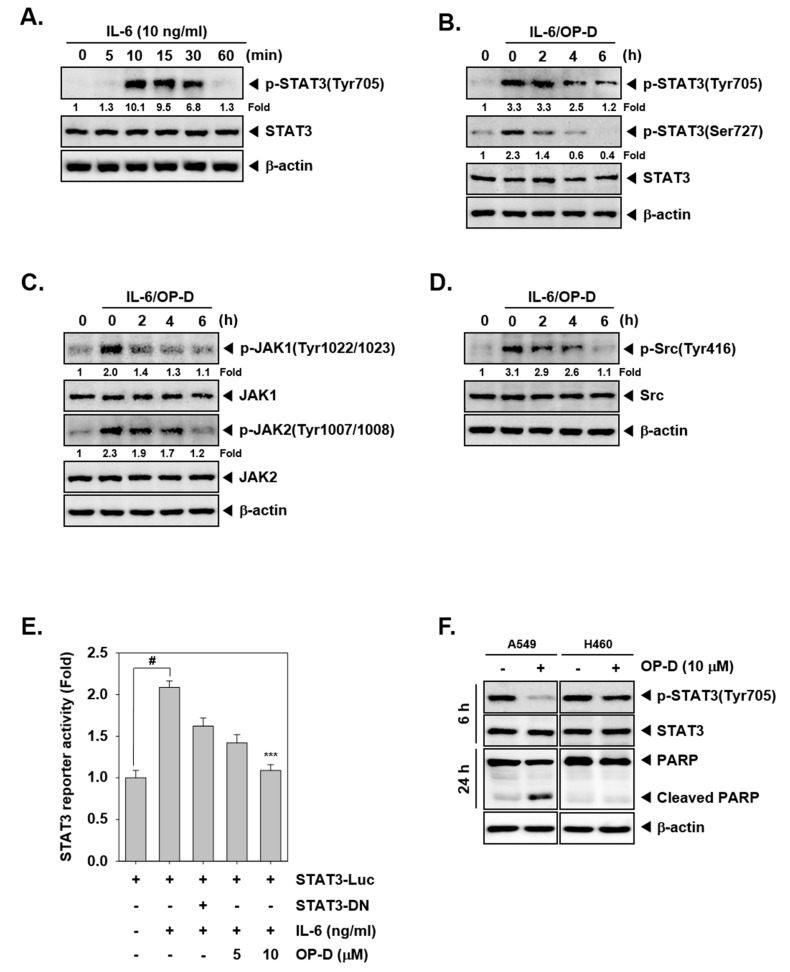
OP-D inhibits IL-6-induced STAT3 activation. (**A**) H1299 cells were treated with IL-6 (10 ng/mL) for the indicated time intervals and Western blot was performed. (**B**–**D**) H1299 cells were treated with 10 µM of OP-D for the indicated times and then stimulated with IL-6 (10 ng/mL) for 15 min and Western blot was done with various proteins. (**E**) H1299 cells were transfected with STAT3-luciferase pretreated with OP-D for 6 h, then simulated with IL-6 and luciferase activity was measured. The results shown are representative of three independent experiments. ^#^ *p* < 0.05; *** *p* < 0.001. Abbreviation: DN = dominant negative. (**F**) A549 and H460 cells were treated with 10 µM of OP-D for 6 h or 24 h and Western blot was performed.

The effect of OP-D on activation of JAK kinases was analyzed next. As shown in Figure 1E, the incubation with OP-D dramatically abrogated the phosphorylation of both JAK1 and JAK2. Interestingly, we also noted that OP-D can also mitigate expression of phospho-Src substantially. We next examined whether OP-D would repress IL-6-stimulated the phosphorylation of STAT3 and upstream kinases. H1299 cells have been known to lack constitutively active STAT3 [16], hence they were used for this experiment. It was observed that inducible phospho-STAT3 level was substantially reduced upon OP-D treatment (Figure 2B). Moreover, IL-6 stimulated JAK1/2 and Src phosphorylation also clearly abrogated upon OP-D exposure (Figure 2C,D).

### 2.3. OP-D Attenuates Reporter Gene Expression and Decreases Levels of STAT3-Regulated Proteins

We analyzed whether OP-D can also regulate STAT3-dependent gene transcription. Interestingly, we noticed that IL-6-induced STAT3 activity was abrogated upon pre-treatment with OP-D (Figure 2E).

### 2.4. OP-D Specifically Inhibits Constitutive STAT3 Activation in A549 Cells, but Not in H460 Cells

We also examined the ability of OP-D to modulate STAT3 activation in human lung cancer cell lines other than A549. As shown in Figure 2F, interestingly we observed that the constitutive activation of STAT3 was suppressed by OP-D in A549 cells, but not in H460 cells. The data suggests that inhibition of STAT3 activation by OP-D may be cell type specific and additional experiments may be required to further corroborate these findings.

### 2.5. OP-D Decreases the Levels of Various STAT3-Regulated Proteins and mRNA Levels

**Figure 3:**
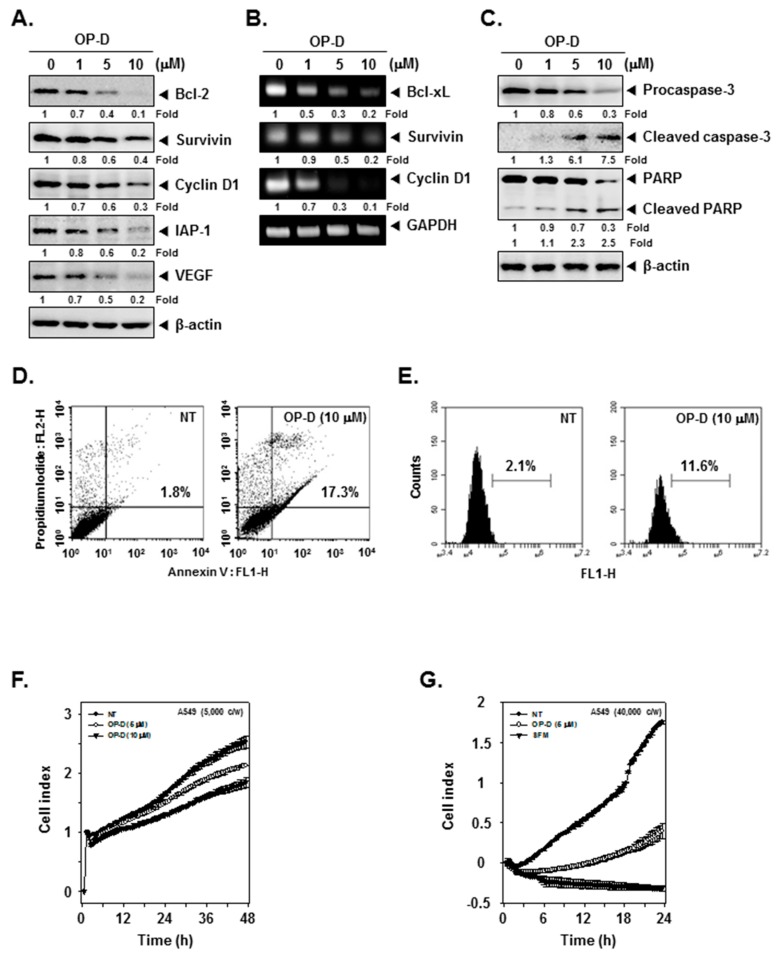
OP-D induces apoptosis, inhibits cell proliferation and invasion. (**A**) A549 cells were treated with the indicated concentrations of OP-D for 24 h and Western blotting was done. (**B**) A549 cells were treated with the indicated concentrations of OP-D for 6 h and reverse transcription-polymerase chain reaction (RT-PCR) was carried out. (**C**) A549 cells were treated as described in panel A and Western blotting was done. (**D**) A549 cells were treated with 10 µM of OP-D for 24 h, stained with FITC (Fluorescein-5-isothiocyanate)-conjugated anti-Annexin V, and flow cytometer was used for analysis. (**E**) A549 cells were treated as described in panel D, fixed, incubated with a TUNEL (terminal deoxynucleotidyl transferase dUTP nick end labeling) reaction solution and analyzed using flow cytometer. (**F**) A549 cells were treated with 5, 10 µM of OP-D and then cell proliferation assay was performed using the Roche xCELLigence Real-Time Cell Analysis (RTCA). (**G**) A549 cells were plated onto a matrigel-coated 16-well CIM-plates, treated with 5 µM of OP-D and then invasion assay was performed using RTCA.

We deciphered the effect of OP-D on various oncogenic proteins and noticed that the expression of anti-apoptotic, cell-cycle-regulator and angiogenic proteins were substantially decreased (Figure 3A). In addition, OP-D treatment also suppressed *Bcl-xL*, *Survivin*, as well as *Cyclin D1* at mRNA levels (Figure 3B).

### 2.6. OP-D Induces Activation of Caspases and Causes Apoptosis

We also analyzed the effect of OP-D on apoptotic markers and found that the drug treatment dramatically induced the expression of active forms of both caspase-3 and PARP (Poly (ADP-ribose) polymerase) (Figure 3C). We next performed flow cytometry based annexin V and TUNEL assays to examine the influence of OP-D on apoptosis. As shown in Figure 3D, apoptosis dramatically increased from 1.8% to 17.3% respectively in cells exposed to OP-D. The results of the TUNEL assay also indicated that the apoptotic population increased from 2.1% to 11.6% after drug exposure (Figure 3E).

### 2.7. OP-D Restrains the Proliferation and Attenuates Invasion of NSCSC Cells

It was found that OP-D treatment could lead to significant reduction in proliferation ability of the cells in a dose- and time-dependent manner (Figure 3F). Interestingly, it was also noted that OP-D treatment could significantly abolish the higher invasive ability of A549 cells (Figure 3G).

### 2.8. Investigation of the Potential Role of Oxidative Stress in OP-D Induced STAT3 Inhibition and Cellular Apoptosis

**Figure 4:**
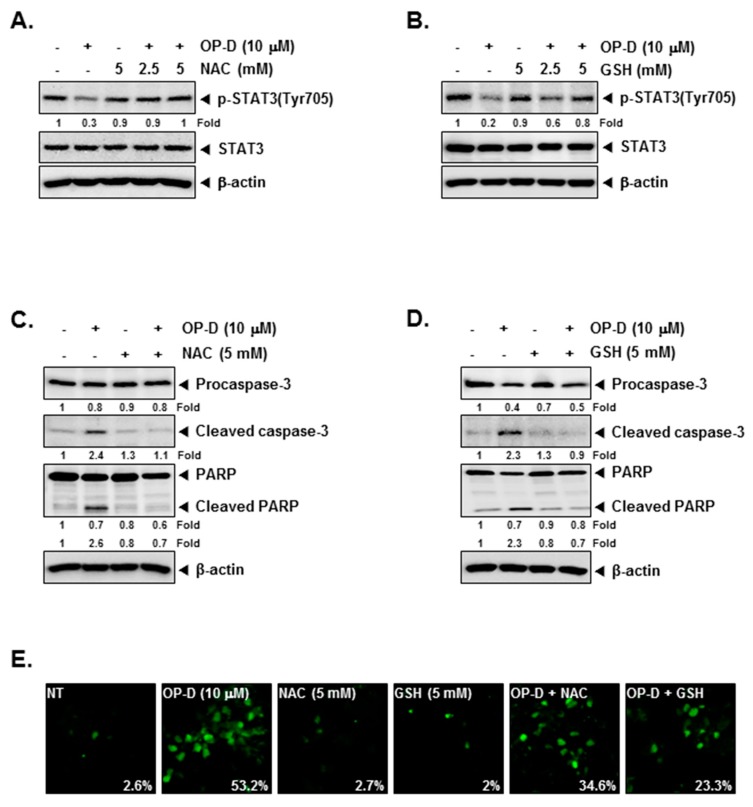
*N*-acetyl-l-cysteine (NAC) and glutathione (GSH) can reverse the STAT3 inhibition. (**A**,**B**) A549 cells were pretreated with indicated concentrations of NAC or GSH for 30 min, and then treated with 10 µM of OP-D for 6 h followed by Western blot analysis. (**C**,**D**) A549 cells were pretreated with 5 mM of NAC or 5 mM of GSH for 30 min, and then exposed to OP-D (10 µM) for 24 h followed by Western blot. (**E**) A549 cells were pretreated with 5 mM of NAC or 5 mM of GSH for 30 min, and then exposed to OP-D (10 µM) for 24 h, harvested, fixed, incubated with a TUNEL reaction solution, and analyzed using a confocal microscope.

**Figure 5:**
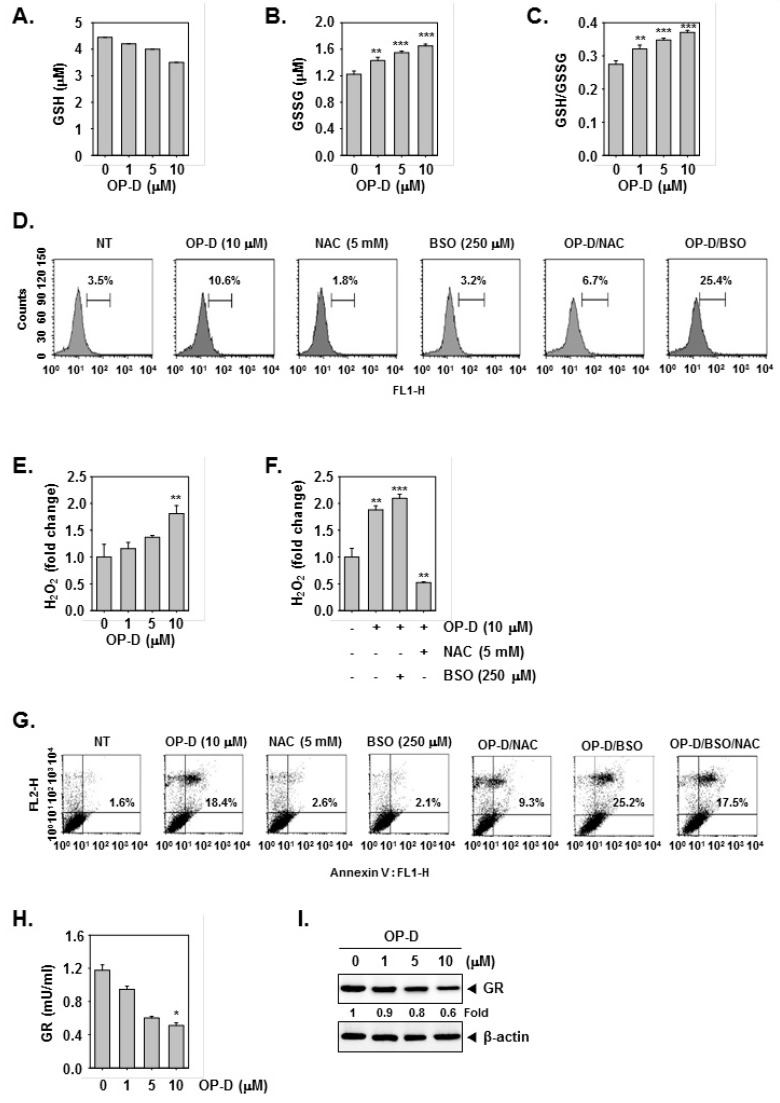
GSH/GSSG imbalance mediates the OP-D-induced ROS production. (**A**–**C**) A549 cells were treated with indicated concentrations of OP-D for 24 h, and then assayed for GSH, GSSG, and GSSG/GSH. (**D**) A549 cells were exposed to NAC (5 mM) with or without buthionine sulfoximine (BSO) (250 µM) for 30 min, and then treated with OP-D (10 µM) for 24 h. The cells were incubated at 37 °C with H_2_DCF-DA for 30 min and analyzed using flow cytometry. (**E**) A549 cells were treated as described in panel **A**–**C** and then subjected to assay for H_2_O_2_ analysis. (**F**) A549 cells were treated as described in panel **D** and then subjected to assay for H_2_O_2_ analysis. (**G**) A549 cells were treated as described in panel D, harvested, stained with FITC-conjugated anti-Annexin V, and analyzed using a flow cytometer. (**H**) A549 cells were treated with the indicated concentrations of OP-D for 24 h, and then collected for investigation using a cellular GR assay kit. (**I**) A549 cells were treated with the indicated concentrations of OP-D for 24 h and Western blotting was performed. Data represent the mean of three measurements ± S.D., * *p* < 0.05; ** *p* < 0.01; *** *p* < 0.001.

*N*-acetyl-l-cysteine (NAC) and glutathione (GSH) are important regulators of oxidative stress [37]. Interestingly, incubation NAC and GSH reversed STAT3 abrogation induced upon drug exposure (Figure 4A,B), Additionally, NAC/GSH pretreatment also attenuated apoptosis induced by OP-D (Figure 4C–E) thereby suggesting the involvement of oxidative stress. The ratio of GSH to oxidized glutathione (GSSG) is a potential marker of oxidative stress [38]. As shown in Figure 5A,B, a significant decrease of GSH and an increase of GSSG levels were noticed. In addition, the GSSG/GSH ratio was observed to also increase (Figure 5C), thereby demonstrating that OP-D can induce oxidative stress. Additionally, OP-D treatment enhanced the level of ROS as evident by flow cytometric analysis. Interestingly, we also noted that pretreatment with *N*-acetyl-l-cysteine (NAC) abolished OP-D induced ROS production, while buthionine sulfoximine (BSO) treatment augmented it (Figure 5D). We next analyzed the H_2_O_2_ levels and as depicted in Figure 5E, a concentration-dependent increase of H_2_O_2_ was noted, that can be attenuated by NAC and enhanced upon BSO exposure (Figure 5F). Interestingly, BSO enhanced OP-D-induced cellular apoptosis, and the increased cell death was also found be attenuated by NAC pretreatment (Figure 5G), thereby providing evidence(s) that GSH/GSSG imbalance may mediate the observed anticancer effects of OP-D. Glutathione reductase (GR) catalyzes the reduction of GSSG to GSH to resist oxidative stress [39] Hence, we measured the effect of OP-D on cellular GR activity and protein level. We found that OP-D can cause a dramatic reduction in both GR activity and protein expression (Figure 5H,I).

### 2.9. OP-D Induces Anti-Tumor Effects in Lung Cancer Preclinical Model and Modulates the Expression of Oncogenic Biomarkers

**Figure 6:**
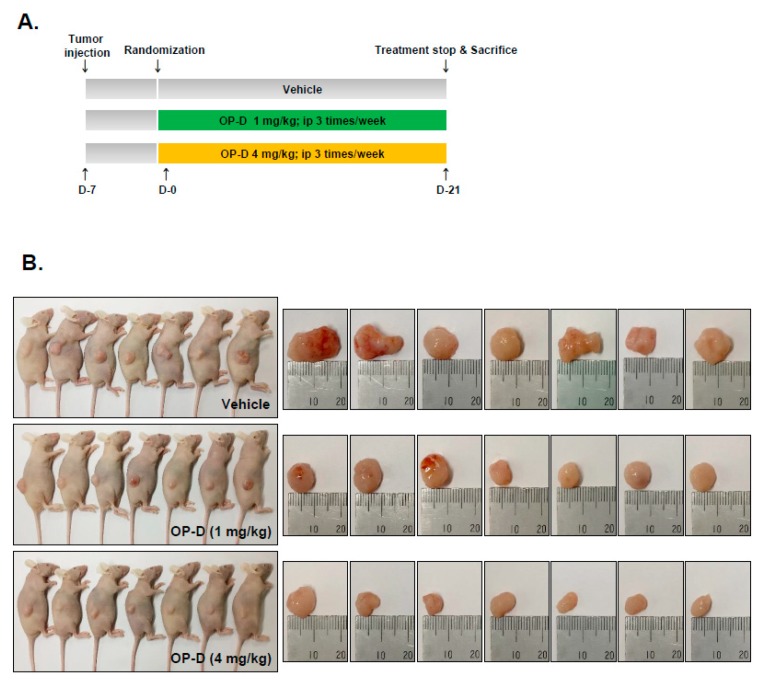

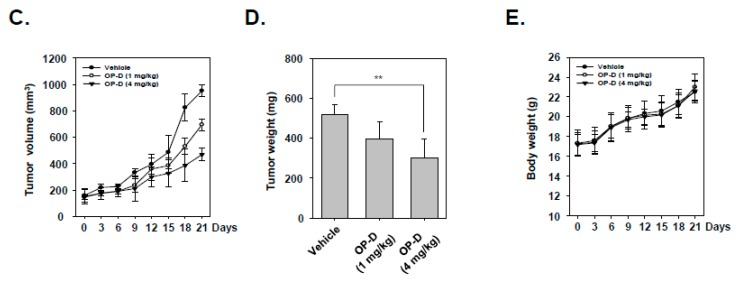
Antitumor effects of OP-D in xenograft mouse model. (**A**) A schematic representation of experimental protocol described in “Materials and Methods.” (**B**) Necropsy photographs of mice bearing subcutaneously implanted with a non-small cell lung carcinoma (NSCLC) tumor. (**C**) Tumor volumes in mouse measured during experiment (n = 7). (**D**) Tumor weight was measured at the end of the experiment. (**E**) Body weight changes of mice were measured at indicated times. Data represent the mean of seven measurements ± S.D., ** *p* < 0.01.

**Figure 7:**
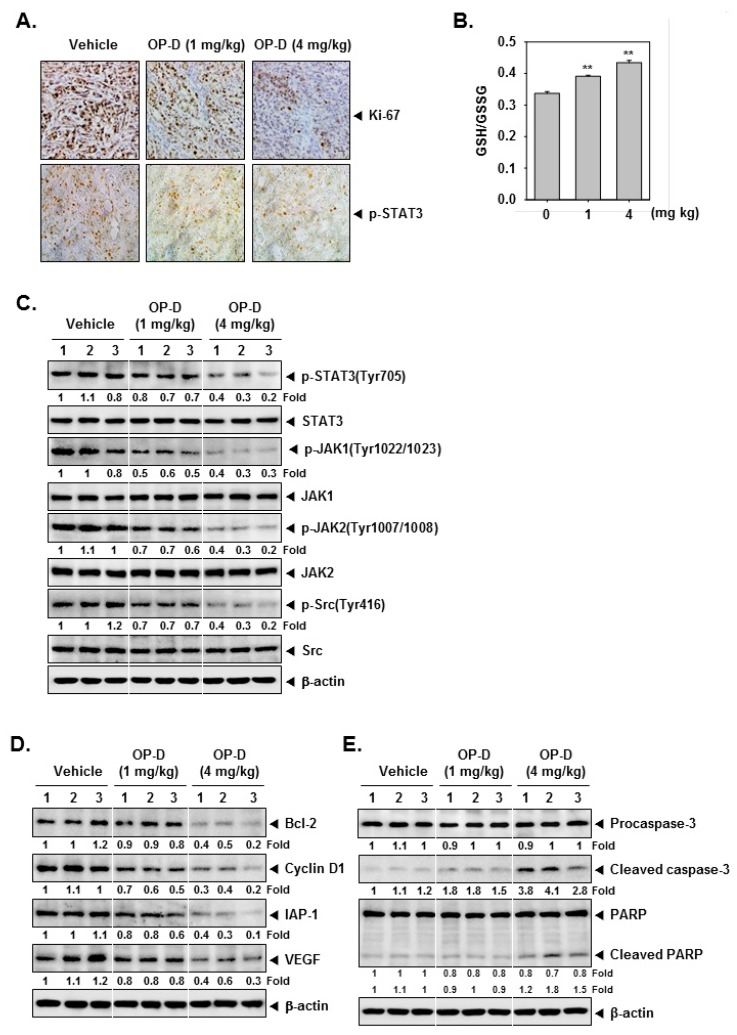
OP-D reduces levels of oncogenic biomarkers in NSCLC tissues. (**A**) Immunohistochemical analysis of proliferation marker Ki-67 and p-STAT3 in NSCLC tumor tissues. (**B**) GSH content in NSCLC tumor tissues treated with OP-D for 3 weeks. (**C**–**E**) Western blot of various proteins of interest was carried out in lysate from vehicle control, and OP-D treated mice. Data represent the mean of three measurements ± S.D., ** *p* < 0.01.

The preclinical effects of OP-D were analyzed as per the protocol depicted in Figure 6A. It was found that the drug treatment caused significant attenuation of the tumor size (Figure 6B), as well as abrogated the tumor growth at day 21 (Figure 6C,D). Interestingly, it also caused an increase in the body weight of the mice (Figure 6E). We also noticed that that OP-D substantially suppressed STAT3 phosphorylation and Li-67 in tumor tissues by immunohistochemistry (Figure 7A, upper and lower panel). Interestingly, a significant decrease of GSH and an increase of GSSG levels were observed in tumor tissues which are in agreement with the findings in cell line (Figure 7B). We also observed that OP-D was effective in suppressing the expression of STAT3, JAK1, JAK2, and Src phosphorylation in tumor tissues (Figure 7C). We further observed that OP-D decreased the expression of diverse proteins (Figure 7D), whereas caspase-3 and PARP activation was increased in tumor tissues (Figure 7E).

## 3. Discussion

In continuation with our efforts to characterize the novel and efficacious blockers of STAT3 activation, this study aimed to investigate the effects of OP-D on the tumor growth in a NSCLC mouse model. We observed that OP-D can exert significant inhibitory effects on STAT3 activation in NSCLC and induce significant oxidative stress that may mediate its observed pleiotropic anti-neoplastic effects.

We first investigated the effect of drug on STAT3 phosphorylation at both tyrosine 705 and serine 727 residues, by Western blot and confirmed these findings by EMSA, immunocytochemistry and luciferase reporter assays. We also examined in detail how OP-D can modulate STAT3 signaling pathway. Identical to STAT3 activation, JAKs can also be activated by the binding of various ligands and cell surface receptors [18]. Activated JAKs phosphorylate tyrosine residues to create binding sites for proteins with the SH2 domain. SH2 domain containing STATs are recruited to the receptor where they are also tyrosine-phosphorylated by JAKs [11,40]. This dual activation of the JAKs/STAT3 cascade can play a pivotal role in tumor progression, and blockade of the JAK/STAT3 signal by OP-D can provide an important therapeutic strategy. The effect of the drug on modulation of STAT3 phosphorylation may also be linked with the inhibition of upstream proteins such as JAK1/2 and c-Src, as we noticed that it can substantially reduce the constitutive activation of these kinases in tumor cells.

STAT3 has also been found to regulate anti-apoptotic functions that can contribute to drug resistance in tumor cells [8,17,22] and it was found that OP-D attenuated the expression of various growth regulatory and cell survival proteins that may contribute to its anti-cancer effects. Interestingly, we noticed that both apoptosis and STAT3 abrogation induced by OP-D can be substantially reversed by antioxidants and thus ROS could play an important role in mediating the anti-cancer effects of OP-D. Indeed, we found that similar to two other STAT3 blockers nimbolide and formononetin previously identified by our group in prostate cancer and multiple myeloma models respectively [17,29], OP-D could also cause a significant increase in ROS (H_2_O_2_ specifically). We also observed that OP-D induced H_2_O_2_ production can be abolished by NAC and augmented upon BSO treatment. Moreover, overexpression of xCT (SLC7A11), a cystine/glutamate antiporter has been recently found to regulate glucose metabolism and intracellular GSH/GSSG redox balance as well as mediate progression of lung cancer [41]. Additionally, it has been reported that drug resistant high-mesenchymal cell state in tumor cells may be mediated through a lipid-peroxidase-dependent pathway that may protect against ferroptosis, which can be induced by both erastin and BSO [42].

Thus, the observed increase in ROS production may be attributed to the imbalance of the GSH/GSSG system and interestingly, GSH reduction was noticed in OP-D treated tumor tissues whereas supplementation of GSH as well as NAC inhibited ROS production and apoptosis, whereas inhibiting GSH synthesis exhibited the opposite effects. Moreover, both the GR activity and expression was found to be modulated upon drug treatment which may suggest that OP-D may primarily exert its STAT3 inhibitory and pro-apoptotic effects via a redox-regulated mechanism in NSCLC cells. Indeed, our group has previously identified two other STAT3 blockers namely nimbolide and formononetin that may function through identical molecular mechanism(s) in prostate cancer and multiple myeloma models respectively [17,29].

Finally, an important aspect of this study was the demonstration of the significant efficacy of OP-D in a preclinical mouse model. Overall, we observed that OP-D could significantly inhibit tumor growth without any apparent side effects as well as substantially reduce expression of p-STAT3 and augment the expression of various pro-apoptotic markers, thereby exhibiting significant anti-proliferative and pro-apoptotic potential.

## 4. Materials and Methods

### 4.1. Reagents

Ophiopogonin D (OP-D, Figure 1A) was purchased from Sichuan Weikeqi Biological Technology Co. Ltd. (Chengdu, China). Stock solution of OP-D (100 mM) was prepared using dimethyl sulfoxide, stored at −80 °C, and diluted in cell culture medium for use. LightShift^®^ Chemiluminescent EMSA kit (Rockford, IL, USA), cell-permeant 2′,7′-dichlorodihydrofluorescein diacetate (H_2_DCF-DA) were obtained from Thermo Fisher Scientific Inc. (Waltham, MA, USA). 5′-biotinylated STAT3 was obtained from Bioneer Corporation (Daejeon, Korea).

### 4.2. Cell Lines

Human lung cancer including A549 cells, H1299, and H460 cells were obtained from the American Type Culture Collection (Manassas, VA, USA). They were grown in RPMI 1640 supplemented with 10% fetal bovine serum (FBS) and 1% penicillin-streptomycin (P/S).

### 4.3. Western Blotting

The Western blot analysis for various proteins of interest was performed as described previously [26].

### 4.4. Electrophoretic Mobility Shift Assay (EMSA) and Immunocytochemistry

DNA binding was analyzed by an electrophoretic mobility shift assay (EMSA) as described before [29]. Immunocytochemistry for STAT3 localization was also performed as described before [29].

### 4.5. STAT3 Luciferase Reporter Assay

H1299 cells were plated in 24-well plates and incubated at 37 °C for one day and luciferase assay was performed as described previously [26].

### 4.6. Reverse Transcription Polymerase Chain Reaction (RT-PCR)

RT-PCR was carried out as described previously [26].

### 4.7. Annexin V and TUNEL Assays

Apoptosis was measured using FITC Annexin V Apoptosis Detection Kit I according to the manufacturer’s protocols. TUNEL staining and assay was done as described previously [26].

### 4.8. Invasion Assay

Roche xCELLigence Real-Time Cell Analyzer (RTCA) DP instrument (Roche Diagnostics GmbH, Mannheim, Germany) was used to measure cellular invasion as described previously [26].

### 4.9. Detection of Reactive Oxygen Species (ROS) and Glutathione Reductase (GR) Determination

Intracellular production of ROS was measured using cell-permeable fluorescent dyes, 5-(and-6)-chloromethyl-2′,7′-dichlorodihydrofluorescein diacetate, acetyl ester (H_2_DCF-DA) as described before [17]. Cellular GR activity was measured with the Glutathione Reductase Assay kit (Abcam) according to the manufacturer’s protocol.

### 4.10. Mice and Housing

All procedures involving animals were reviewed and approved by Kyung Hee University Institutional Animal Care and Use committee [KHUASP(SE)-17-046]. Five-week-old athymic balb/c *nu/nu* female mice were purchased from Nara biotech (Seoul, Korea). Subcutaneous implantation of was performed as described previously [16].

### 4.11. Experimental Protocol

When the tumors had reached 0.25 cm in diameter, the mice were randomized into the following groups (n = 7/group) based on the tumor volume. Group I (control) was treated with PBS (100 µL; i.p.; 3 times/week), group II was treated with OP-D (1 mg/kg; i.p.; 3 times/week), and group III was treated with OP-D (4 mg/kg; i.p.; 3 times/week) (n = 7). The tumor volume was measured, and tissues were processed thereafter as described before [16].

### 4.12. Statistical Analysis

Data are expressed as the mean ± S.D. In all figures, vertical error bars denote the S.D. The significance of differences between groups was evaluated by Student’s *t*-test and one way analysis of variance (ANOVA) test. A *p* value of less than 0.05 was considered statistically significant.

## 5. Conclusions

We report here that OP-D can function as a pharmacological inhibitor of STAT3 signaling and may provide novel therapeutic options for NSCLC patients in future.

## Abbreviations

STAT3	Signal transducer and activator of transcription 3
JAK	Janus kinase
Src	Proto-oncogene tyrosine-protein kinase
NSCLC	Non-small cell lung carcinoma
ROS	Reactive oxygen species
NAC	*N*-acetyl-l-cysteine
GSH	Glutathione
BSO	Buthionine sulfoximine
GR	Glutathione reductase
FBS	Fetal bovine serum
PFA	Paraformaldehyde
TUNEL	Terminal deoxynucleotidyl transferase dUTP nick end labeling
i.p.	Intraperitoneal injection
